# Novel feed additive delivers antimicrobial copper and influences fecal microbiota in pigs

**DOI:** 10.1128/spectrum.04280-23

**Published:** 2024-04-17

**Authors:** Mariana Fernandez, Jonathan Thompson, Alexandra Calle

**Affiliations:** 1 Texas Tech University, School of Veterinary Medicine, Amarillo, Texas, USA; Agriculture and Agri-Food Canada, Lacombe, Canada

**Keywords:** copper, alginate, antimicrobial, slow release, encapsulation, time release, pathogen shedding, microbiome, preharvest intervention

## Abstract

Dehydrated alginate beads formulated with copper were synthesized and tested as a feed additive to influence the microbiota in finishing pigs and potentially use them as a preharvest intervention to reduce fecal pathogen shedding. The efficacy of the copper beads was tested *in vitro* and *in vivo. In vitro*, *Salmonella* was significantly (*P* < 0.05) reduced when in contact with the copper beads solution for up to 6 h, with a 5.4 log CFU/mL reduction over the first hour. Chemical analysis of the soak solutions demonstrated the beads delivered their copper payload gradually over the same period the bactericidal effect was observed. For the *in vivo* experiments, pigs (*n* = 48) supplemented with the copper beads experienced significant shifts in their microbiota. Enterobacteriaceae (EB) increased by 1.07 log CFU/g (*P* < 0.05), while lactic acid bacteria (LAB) decreased by 1.22 log CFU/g (*P* < 0.05) during the treatment period. When beads were removed from the feed, EB and LAB concentrations returned to baseline, indicating copper beads led to measurable and significant changes in microbial loads. Fecal microbiome analysis conducted to explore additional changes by copper bead supplementation demonstrated that, at the phylum level, there was an increase in Firmicutes, Euryarchaeota, and Acidobacteriota, while at the genus level, an increase in *Methanosphaera* and *Pseudomonas* was observed. Measures of copper in swine feces showed values ~20 times higher in the treatment group than in the control group during the treatment period, suggesting that dehydrated alginate copper beads were effective in delivering antimicrobial copper to the animal hindgut.

**IMPORTANCE:**

Copper has long been known to have antimicrobial properties. However, when water-soluble salts are fed to livestock, the copper may rapidly dissolve in gastric contents and fail to reach the gut. Here, specially formulated copper beads are seamlessly incorporated into feed and allow copper to remain longer in the gastrointestinal tract of animals, reach deep into both the foregut and hindgut, and shift microbial populations. The technology delivers antimicrobial copper to the animal hindgut and potentially reduces pathogenic microorganisms before animal slaughter.

## INTRODUCTION

Copper (Cu) has been historically used during swine production due to its health benefits, including growth promotion, animal health prevention, and control of microbial populations in the animals’ gastrointestinal (GI) tract. The utilization of copper as a supplement for enhancing animal growth performance has been the subject of extensive research, with compelling findings indicating its positive impact on factors such as increased lipase activity and growth hormone secretion (1–3). As an intervention for microbial control, copper is particularly relevant in swine production since pigs tolerate high levels of the mineral in their diets.

The GI tract flora plays an important role in safeguarding both animal and public health. Reducing pathogen levels in the GI tract is a recommended practice for minimizing the risk of microbial transmission to food during post-harvest production activities (4, 5). Various strategies are presently employed in animal production, often as prophylactic measures. These encompass the use of antibiotics, vaccines, direct-fed microbials, and antimicrobials in drinking water. Each strategy carries its own set of advantages and disadvantages.

In the case of antibiotics, the risk of the development of antimicrobial-resistant organisms exists (6). Thus, many countries have enacted legislation limiting the presence of antibiotic residues in food. On the other hand, disinfectants such as sodium chlorate can be applied in drinking water to kill GI tract bacteria in pigs; however, its effect tends to be reduced to only for a specific group of organisms (7, 8). Direct-fed microbials inhibit pathogens by competitive exclusion and may enhance weight gain (9–11). This approach inoculates animals with high levels of probiotic bacteria to starve pathogens of nutrients; nonetheless, long-lasting efficacy of the approach has been questioned (12).

Cu is a known antimicrobial agent that non-specifically targets bacteria, viruses, and fungi (13). In 2008, copper was recognized by the U.S. Environmental Protection Agency as the first metallic antimicrobial agent. This led to many studies on the various properties of copper as an antimicrobial (14). Early studies demonstrated that metallic Cu and Cu alloy surfaces can reduce *Salmonella* and *Escherichia coli* O157:H7 concentrations and inhibit biofilm formation compared to other surfaces used in food processing facilities (15, 16). It has also been used in the food industry to reduce spoilage microorganisms and control foodborne pathogens (17).

The bacteriostatic and bactericidal properties of copper are thought to reduce inflammation, which can lead to growth promotion in animals (18). The nutritional requirement for Cu in feed is 5–10 mg/kg of diet; however, as much as 75–250 mg/kg can be supplemented as growth promotant in swine (18). However, long-term feeding of copper as a supplement or in a basal diet can reduce its growth-promoting effects since copper can accumulate in the tissues of animals and cause copper toxicity (19). Early research suggests that copper supplementation at higher doses of >250 mg/kg can be harmful to the animals as it can accumulate in the liver and kidney (20, 21). Therefore, a method for copper delivery that decreases the rate of copper absorption and provides targeted delivery deep in the GI tract is needed.

One strategy gaining interest in the scientific community is using hydrogel beads as delivery agents in animal diets (22). Hydrogels are cross-linked three-dimensional (3D) network structures formed from hydrophilic polymers that provide a physical barrier and 3D structure while remaining biodegradable (23, 24). Furthermore, when hydrogels are dehydrated, the release of the substance being delivered is slowed (25).

Considering the potential benefits of supplementing pigs’ diets with dehydrated copper alginate beads (CBs), this study sought to investigate a mechanism that guaranteed long-lasting delivery of copper in the GI tract as a dietary supplement for swine and assessed its effect in fecal bacterial populations.

Specifically, this study evaluated several hypotheses: (i) dehydrated alginate beads cross-linked by copper cations will provide a slow release of copper ions to the surroundings as the hydrogel rehydrates and decomposes; (ii) the slow leakage of copper from beads will facilitate the metal to remain longer in the swine GI tract; (iii) dehydrated alginate beads cross-linked by copper cations will reduce *Salmonella* populations *in vitro* owing to the antimicrobial effects of copper ion; and (iv) the supplementation of copper beads will produce changes in the swine GI tract microbiota, reflected in the observable shift of bacterial populations in feces.

## MATERIALS AND METHODS

### Preparation of CBs

All ingredients used to prepare the CB are considered “generally recognized as safe” by the Food and Drug Administration (FDA), which means they can be used safely for consumption by humans and animals (26). The beads were prepared using a modified method of Levy and Edwards-Levy as a guide (27). In brief, a bath solution containing copper was prepared by mixing distilled water and copper sulfate pentahydrate to achieve a 10% m/m copper sulfate solution (LabChem Inc, Zelienople, PA, USA). The solution was made in a 4-L glass beaker and was placed on a magnetic stirrer hot plate to homogenize the mixture. Then, a second solution containing 1% m/vol alginic acid (FitLane Nutriton), plus 2% m/vol pectin (Modernist Pantry, Eliot, ME, USA), plus 3% m/vol collagen peptides (Orgain, Irvine, CA, USA) was prepared in distilled water on a combination stir/hot plate. The addition of pectin and collagen peptides aided in the formation of a protective membrane on the beads upon alkalization. The membrane is formed as the protein directly binds to a polysaccharide through amide linkages in a transacylation reaction. The reaction creates a series of amide cross-links between molecules to form a complex network that holds the bead together (27).

The second solution was added to the copper-containing bath solution dropwise, which instantly formed alginate copper hydrogel beads of approximately 4 mm in diameter. The formed hydrogel beads remained in the copper bath solution for a minimum of 30 min, strained, and then washed in distilled water. After washing with distilled water, beads were placed in a water bath, and 2–3 mL of sodium hydroxide was added dropwise while stirring to raise the pH, which initiated the transacylation reaction described in the work of Levy and Levy-Edwards. The solution and beads turned a characteristic deep blue as copper binds proteins at basic pH to form the Biuret reagent. Then, a dilute hydrochloric acid was used to neutralize the solution. The beads were washed and drained with deionized water three times. Subsequently, the beads were placed on a plastic tray for drying in an incubator at 72°C for approximately 24 h. As the beads dried, their volume was dramatically reduced, and small deep-green granules resulted. The copper content in CB was measured using atomic spectroscopy in preliminary experiments and was estimated to be 12% by mass. Dosages fed to the pigs were calculated as 23 g of CB per 2.5 kg of feed. The “*in vitro* experiments” further describe the details.

### *In vitro* experiments

#### Microbial reduction assay

*Salmonella* strains ATCC 31194, ATCC 6962, and ATCC 8398 were used in this study. Cultures were maintained frozen at −80°C and, before the experiment, a full loop was transferred to tryptic soy broth (TSB) (Remel, San Diego, CA, USA) and incubated at 37°C for 24 h. Each strain was grown individually, and upon incubation, the three strains were combined to make a *Salmonella* cocktail by mixing 1 mL from each into a sterile test tube. Serial dilutions from this cocktail were performed in buffered peptone water (BPW) (EDM, Darmstadt, Germany) to reach a concentration of ca. 10^6^ CFU/mL. The concentration was verified by conventional bacterial enumeration, using a spread plating technique on tryptic soy agar (TSA) (Oxoid, Basingstoke, Hampshire).

Test tubes containing each 5 mL of the bacterial cocktail were used to test the antimicrobial effect of CB on *Salmonella*. One tube was treated with 1 g of CB, and a control tube remained untreated. The antimicrobial effect of CB was observed over time. The tubes were kept for 6 h under agitation, and every hour, the aqueous portion containing the bacterial solution was extracted (leaving the remaining CB at the bottom) and replaced with a new freshly made bacterial cocktail (ca. 10^6^ CFU/mL). The solution removed each hour was split and tested for microbial reduction and copper concentration. *Salmonella* was enumerated by spread plating on TSA, and copper concentration was evaluated by atomic absorption spectroscopy (described below). The purpose of leaving the CB in the test tube was to test the ability of CB to continue to release copper and their effect on reducing *Salmonella* over time. This experiment was conducted in triplicate.

#### Copper concentration in microbial reduction assay

Upon removal of the bacterial cocktail every hour, as described above, an aliquot was used to test the copper concentration remaining in the bacterial solution. All samples were digested in nitric acid, and copper concentration within the digest was determined by flame atomic absorption spectroscopy using the 324.8-nm line on a Shimadzu AA-6300 instrument using acetylene/air flame. In brief, external copper standards were prepared using copper sulfate salt and used to construct a calibration line. The concentration of copper present in the sample was computed using the calibration and converted to parts per million (ppm) of copper present in the 1-mL sample volume.

### *In vitro* experiments

#### Experimental design

**Fig 1:**
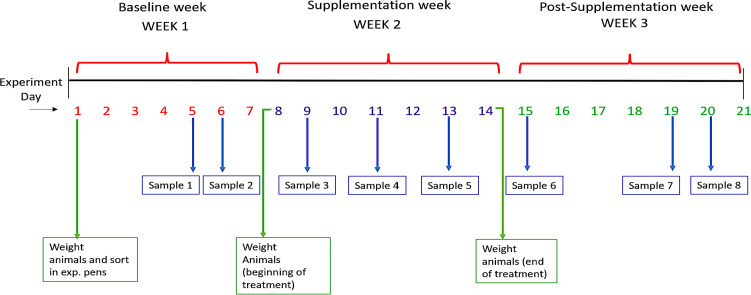
Timeline of *in vivo* experiment. The three experimental weeks can be seen at the top of the figure. Experiments were carried out over 21 days. The specific days for sample collection and recording the weight of the animals are indicated in the lower portion of the figure.

Animal experiments were conducted at the Texas Tech University New Deal farm using 48 6-month-old pigs finishing pigs (PIC Camborough). The experimental procedure was reviewed and approved by the Texas Tech University Institutional Animal Care and Use Committee (IACUC) (IACUC-approved protocol #T21079). The National Research Council nutritional requirements were followed for the daily standard animal feed (80.14% corn, 17% soybean meal, 1% yellow grease, 0.85% dicalcium phosphate, 0.6% limestone, 0.13% trace minerals, 0.15% salt, and 0.13% vitamin premix), and water was available at all times. Pig selection for this study was based on weight and sex (24 male and 24 female). Before the study, the pigs were classified by weight under low weight (between 100 and 130 lb or between 45.5 and 59.0 kg) and high weight (>130 lb or 59 kg). Average weight and standard deviations were 118.2 ± 8.6 lb and 144.6 ± 9.2 lb (53.7 ± 3.9 kg and 72.3 ± 4.6 kg) for the groups, respectively. Pigs were then randomly assigned to either a treatment (TRT) or control (CTL) pen with an equal number of pigs in the low-weight and high-weight categories in each pen. Pen was considered the experimental unit. Animals were moved to their corresponding experimental pens 1 week prior to the experiments to allow for acclimation. Thus, 48 pigs were assigned to eight pens (four TRT and four CTL pens) containing six animals (three male and three female). The application/supplementation of CB to the treatment pens occurred only during the second week of the study. Figure 1 outlines the treatment calendar.

This 21-day study was divided into 3 weeks: (i) baseline week (experiment days 1–7), (ii) supplementation week (experiment days 8–14), and (iii) post-supplementation week (experiment days 15–21) (Fig. 1). The dehydrated CBs were supplemented to the four TRT group pens, and no beads were supplemented to the four CTL group pens. The CB treatment was applied at the pen level by mixing the beads with the feed daily. During the first 7 days (baseline week), feed intake information was assessed daily by placing a known amount of feed and weighing the remaining feed the next day. Estimates indicate that animals consume an average of 2.5 kg of feed/day. A dose of 23 g of CB was added to each 2.5 kg of feed (copper content in CB has been estimated in prior experiments to be 12% by mass), estimating a supplementation of 2.7 g of copper daily, which is equivalent to ca. 440 ppm of copper/kg of feed. Separate chemical analysis of swine feed without the addition of CB indicated a baseline copper concentration of only ~20 ppm. Animals consumed feed supplemented with CB without bias.

#### Fecal sample collection

Figure 1 defines fecal sampling days during the baseline, supplementation, and post-supplementation periods. Feces were collected on experiment days 5 and 6 (baseline week when no CB treatment was administered); days 9, 11, and 13 (supplementation week); and on days 15, 19, and 20 (post-supplementation, when CB was removed from feed). This sequence allowed the evaluation of microbial changes and copper concentration prior to, during, and after CB supplementation. On each sampling occasion, approximately 10 g of fecal samples was manually obtained directly from the rectum using sterile gloves. Samples were placed in specimen cups, labeled, and transported immediately in a cooler with ice packs to the Texas Tech University food microbiology lab for further microbial analysis.

#### Enterobacteriaceae and lactic acid bacteria enumeration

Enterobacteriaceae (EB) and lactic acid bacteria (LAB) were used as indicators of microbial shift influenced by the treatment. From each sample, 10 g of feces was placed in a filtered Whirl-Pak bag (Whirl-Pak, Madison, WI, USA), combined with 90 mL of TSB, and homogenized for 2 min. Serial 10-fold dilutions were prepared by placing 1 mL of the homogenate in 9-mL tubes of BPW.

For the enumeration of EB, 1 mL of each dilution was plated in duplicate on 3M Petrifilm Enterobacteriaceae count plates (3M, St. Paul, MN, USA) and incubated for 24 h at 37°C (as indicated in AOAC Official Method 2003.01). For the enumeration of LAB, recommended steps were followed from the *FDA Bacteriological Analytical Manual*. An aliquot of 100 µL of each dilution was spread plated in duplicate on De Man, Rogosa, and Sharpe agar (Neogen Culture Media, Lansing, MI, USA) and incubated for 72 h at 37°C (28). Upon incubation, colonies were enumerated, reported as CFU per gram, and log-transformed for statistical analysis.

#### Copper concentration in fecal samples

Feces from randomly selected pigs (TRT and CTL) obtained during the supplementation and post-supplementation weeks were tested using atomic spectroscopy. Samples were dried overnight at 70°C in uncapped vials within a drying oven before being allowed to cool to room temperature. The mass of dried feces plus vials was recorded, and the mass of empty vials was noted separately. Fecal matter mass was found by difference. Dried feces were digested stepwise in concentrated nitric acid. A volume of 2 mL of concentrated nitric acid was added to each uncapped vial and allowed to react for 24 h within a fume hood. The resultant aliquot was transferred to a 20-mL scintillation vial, and an additional 2 mL of nitric acid was used to rinse the sample tube and promote the quantitative transfer of copper. The resulting volume (ca. 4 mL) remained within a scintillation vial, was capped, and placed in an oven at 70°C for an additional 6 h. During this period, vials were occasionally vented in a fume hood due to the production of brown, gaseous oxides of nitrogen. Then, 16 mL of reverse osmosis purified water was added to each vial. The vials were returned to the oven at 70°C overnight to finalize sample digestion. Copper concentration within the digest was determined by flame atomic absorption spectroscopy using the 324.8-nm line on a Shimadzu AA-6300 instrument using acetylene/air flame. Copper standards were prepared using copper sulfate salt and used to construct a calibration line. The calibration was linear over the range of 0–8 ppm with *R*^2^ = 0.9994. The concentration of copper present in the sample was computed using the calibration and converted to mass of copper in the 20-mL sample volume, and this amount was normalized to dry mass of feces for reporting.

#### Microbiome analysis

A total of 30 fecal samples were selected over the 21 experimental days for microbiome analysis (equal number from TRT and CTL groups). DNA was extracted from feces using QIAamp PowerFecal Pro DNA Kit (Qiagen, Germantown, MD, USA) according to the manufacturer’s protocol and quantified using a NanoDrop (Thermo Fisher Scientific, Wilmington, DE, USA). Hi-throughput V3–V4 16s rRNA sequencing was performed on the Illumina MiSeq platform. The amplicon was sequenced on Illumina paired-end platform to generate 250-bp paired-end raw reads, then merged and pretreated to obtain clean tags. The clean tags were then detected and removed to obtain effective tags, which were used for subsequent analysis.

#### Statistical analyses

Statistical analyses were performed using R (version 4.2). For the *in vitro* experiment, *Salmonella* recovered after exposure to the CB at each hour was assessed. Considering that an untreated cocktail was added at each hour to the same beads, a pairwise *t*-test adjusted by the Holm method was used to assess *Salmonella* reduction after each hour of exposure to the treatment. For the *in vivo* experiments, a power analysis was conducted using G*Power (version 3.1.9.7) to determine the minimum sample size required to test the hypothesis that supplementation with CB will alter microbial communities in fecal samples. Results indicate that to achieve 90% power, at a significance criterion of α = 0.05, *n* = 40. Thus, the obtained sample size of *n* = 48 was adequate to test the hypothesis. The nature of the data in the *in vivo* experiments is repeated measurement of bacterial counts and weight over time, depending on treatment types. For this analysis, a repeated measure analysis of variance (ANOVA) was used to account for a possible correlation between bacteria counts and weight over time. For the ANOVA, standard normality assumptions were explored and met. For each statistically significant interaction effect between the treatment groups and time, a post hoc statistical comparison between each group adjusted using the Bonferroni correction was performed. For microbiome analyses, a permutational multivariate analysis of variance (using Bray-Curtis distance matrix) was performed to assess groups that shifted in the swine fecal microbiome. Statistically significant differences were considered at *P* < 0.05.

## RESULTS

### *In vitro* experiments

#### *Salmonella* reduction by copper beads

**Fig 2:**
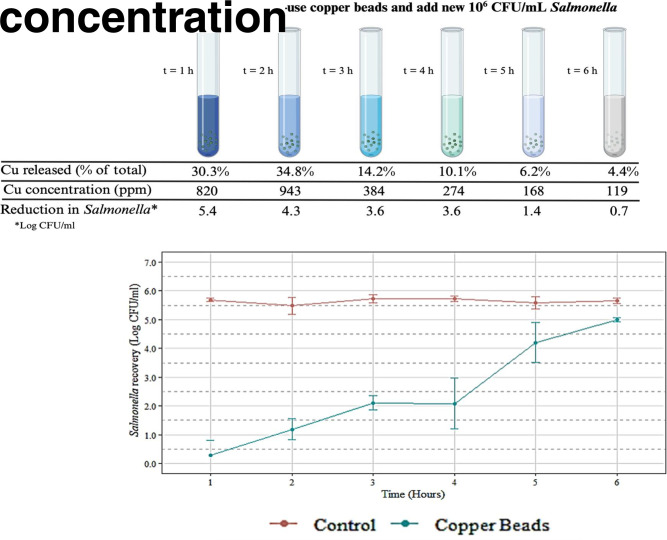
(Top) Schematic of the experiment and the corresponding measurements each hour. Time (*t*) corresponds to hours during which the CBs were tested. (Middle) Percent copper released during each time interval, resulting solution pH, and observed reduction in *Salmonella*. (Bottom) *Salmonella* recovery after exposure from 1 to 6 h to copper beads (copper beads, *n* = 18) or no treatment (control, *n* = 18).

Reduction of *Salmonella* after treatment with the slow-release dehydrated copper beads was tested. Microbial reduction was measured every hour for 6 h. As represented in Fig. 2, the CB treatment significantly (*P* < 0.05) reduced the *Salmonella* concentration during the first 5 h compared to the control. A dramatic reduction of 5.4 log CFU/g was observed after the first hour of treatment. Moreover, each consecutive hour when a new load of *Salmonella* was added to the CB, the residual copper continued destroying the organism, as observed in the subsequent reductions of 4.29, 3.61, 3.64, 1.38, and 0.67 log CFU/g every hour until the sixth hour. These results suggest that *in vitro*, when copper is encapsulated in the form of dehydrated beads, it continuously delivers and maintains the antimicrobial effect of copper to act as a bactericidal agent over time.

#### Copper release over time

To evaluate the time-release property of the CB, copper concentration was measured each hour over 6 h during the microbial reduction assay described above. The pH of the solution before the addition of CB was 6.95 ± 0.03. As depicted in Fig. 2, the pH of the solution dropped when the CBs were added and increased again over time. On the other hand, when copper was measured after the first hour, 30.3% of the copper in the CB was released into the bacterial solution. Thereafter, copper continued being released (34.8, 14.2, 10.1, 6.2, and 4.4%) into the solution with CB throughout the 6-h experiment. Recall that only the bacterial solution in the tube was replaced each hour. These results demonstrate that copper, when encapsulated in the beads, is slowly released into the solution, and its effectiveness remains over time.

### *In vivo* experiments

#### Animal performance with CB supplementation

Pigs were weighed three times during the experiment to survey any differences in performance (weight fluctuations) over the course of supplementation. Weights were obtained before CB supplementation and two times during supplementation (first day of CB and last day of CB). No significant differences (*P* > 0.05) were found between the TRT and CTL groups on weight gain (Table 1), which means copper bead supplementation did not affect the performance of the pigs demonstrated by the normal weight gain with the predetermined diet in finishing or near-market weight pigs over a 1-week period. No adverse health effects of CB supplementation were noted.

**TABLE 1:** Weight of pigs throughout experiment^*c*^

Week	Experiment day	Treatment group^*a*^	Weight (kg)^*b*^
Baseline	1	TRT	70.54 ± 13.14
Baseline	1	CTL	73.77 ± 8.71
Supplementation	8	TRT	83.99 ± 19.34
Supplementation	8	CTL	83.26 ± 14.32
Supplementation	14	TRT	80.04 ± 10.9
Supplementation	14	CTL	78.67 ± 14.51

^*a*^ Represents groups in study (TRT = animals fed CB, *n* = 24; CTL = animals not fed CB, *n* = 24).

^*b*^ Weight in kilogram ± standard deviation.

^*c*^ No statistical differences observed (baseline *P* = 0.35; supplementation experiment day 8, *P* = 0.71; supplementation experiment day 14, *P* = 0.89) between TRT and CTL groups.

#### Enterobacteriaceae and lactic acid bacteria enumeration

**Fig 3:**
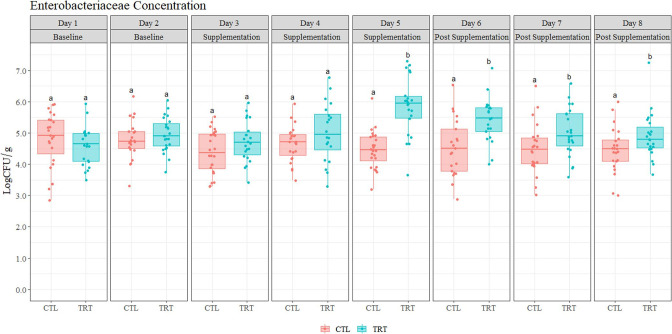
Enterobacteriaceae enumeration from fecal samples (*n* = 361). Animals in the treatment (TRT) group were supplemented with copper beads, and animals in the control (CTL) group were not supplemented with copper beads. Significant statistical differences (*P* < 0.05) are represented with different lowercase letters (e.g., a and b). The first row lists each consecutive sampling day throughout the duration of the experiments. The row below indicates whether supplementation occurred (or not) on the sampling day indicated.

As represented in Fig. 1, fecal samples were collected at eight sampling points before, during, and post-treatment to assess the effect of CB on bacterial shedding when fed to the pigs. Bacterial enumeration indicated a shift in microbial loads of EB and LAB during supplementation. Results from the repeated measures ANOVA show that treatment (*P* = 0.012), day or exposure time (*P* = 0.022), and the interaction between treatment and day (*P* = 0.012) influenced concentrations of EB. The change in EB concentrations in the TRT group from baseline (experiment day 5, first sampling day) until the end of supplementation (experiment day 13, fifth sampling day) corresponded to a 1.07 log CFU/g increase, which was significantly higher (*P* < 0.05) relative to the baseline. On the other hand, EB concentration in the CTL group did not change between sampling days (*P* > 0.05), indicating that the increase in EB was attributed to the copper treatment. Concentrations of EB continued to increase even after CB had been removed for 1 day (day 15, sixth sampling day). Furthermore, a reduction in EB concentrations was observed at experiment days 19 and 20, indicating that concentrations of EB returned to baseline (*P* > 0.05) after 4 days of CB removal (Fig. 3).

**Fig 4:**
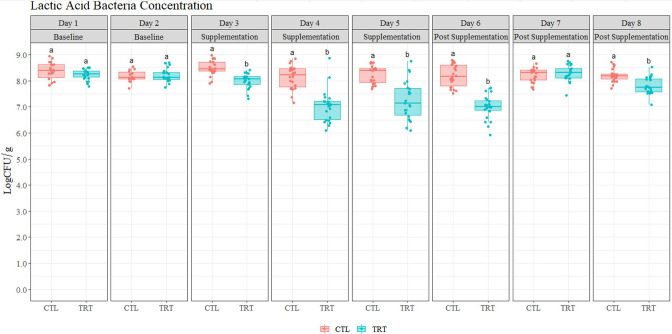
Lab enumeration from fecal samples (*n* = 356). Animals in the treatment (TRT) group were supplemented with copper beads, and animals in the control (CTL) group were not supplemented with copper beads. Significant statistical differences (*P* < 0.05) are represented with different lower case letters (e.g., a and b). The first row lists each consecutive sampling day throughout the duration of the experiments. The row below indicates whether supplementation occurred (or not) on the sampling day indicated.

With respect to LAB enumeration in fecal samples before, during, and after supplementation, data obtained from the repeated measures ANOVA indicated that treatment (*P* = 6.67e-8), day or exposure time (*P* = 1.22e-11), and the interaction between treatment and day (*P* = 1.08e-09) also had an effect on concentrations of these microorganisms. As can be observed in Fig. 4, post hoc analyses resulted in significant differences (*P* < 0.05) between TRT and CTL (decrease of 0.52 log CFU/g) after only 1 day of CB supplementation, which persisted up to 5 days after CBs were removed. Similarly, as in EB, LAB concentrations in the CTL group did not change between sampling days (*P* > 0.05), indicating that the decrease observed can be attributed to the copper treatment. The change in LAB concentrations in the TRT group from baseline (first sampling day) until the end of supplementation (day 13, fifth sampling day) corresponded to a 1 log CFU/g decrease, which was significantly lower (*P* < 0.05) from the baseline. Interestingly, significant differences (*P* < 0.05) were observed on experiment day 20, which was 6 days after the beads had been removed. Supplementation with CB significantly reduced counts of LAB in swine fecal samples, indicating this class of organism is susceptible.

#### Copper concentration in feces

**Fig 5:**
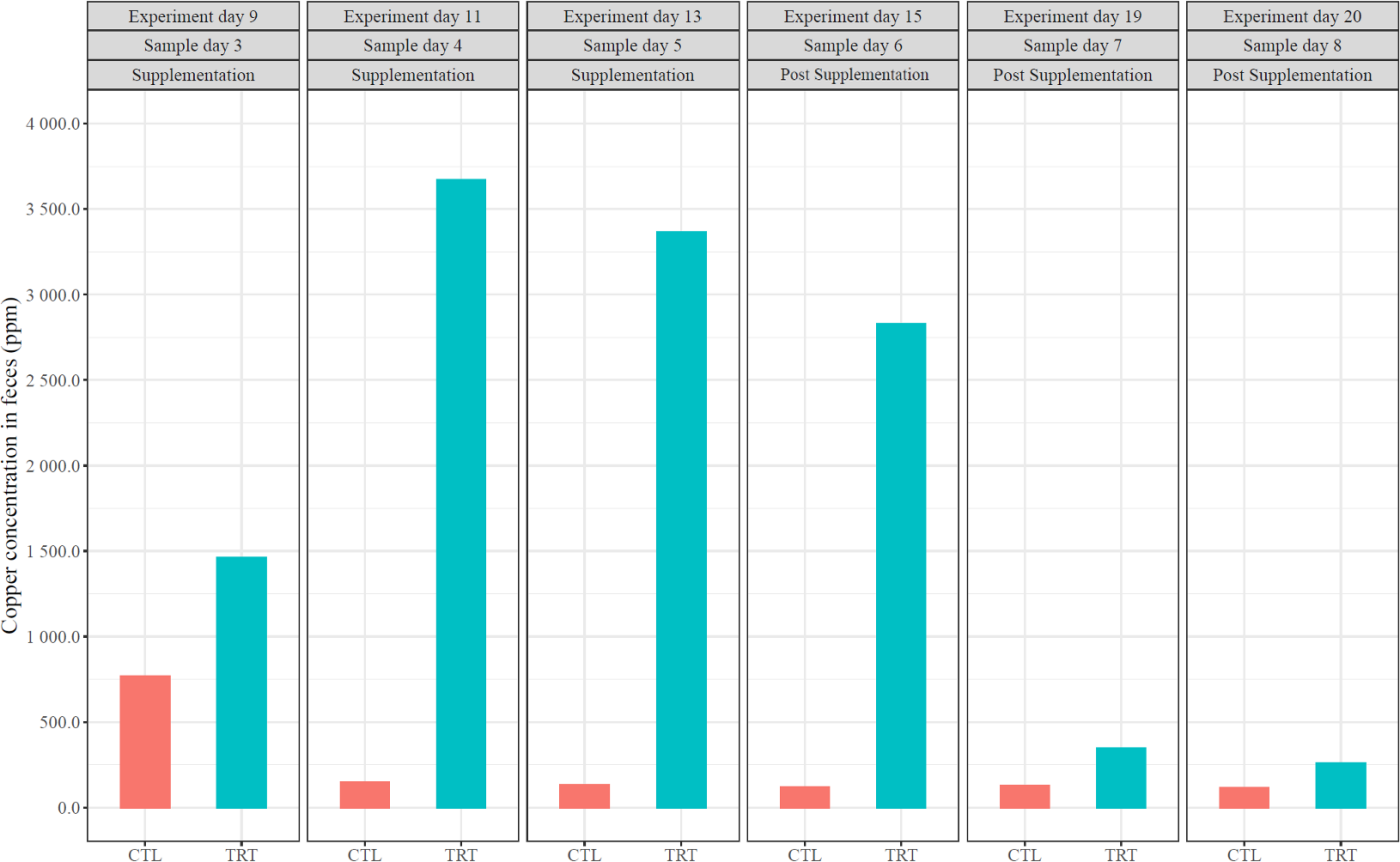
Concentration of copper in feces (*n* = 11 TRT, *n* = 11 CTL). Animals in the TRT group were supplemented with copper beads, and animals in the CTL group were not supplemented with copper beads. At the top of the graph, the first row, or “experiment day,” represents each consecutive day throughout the duration of the experiments. The second row, or “sample,” represents each day fecal samples were collected throughout the experiment. The third row describes when the supplement was applied.

Copper concentrations in feces from the CTL group remained relatively steady throughout the experiment. Contrastingly, the copper concentration in the TRT group was 23.4 times greater than in the CTL samples (3,433 ppm in TRT and 147 ppm in CTL) on experiment day 11 during supplementation. Once the beads were removed from feed, a downward trend in copper concentration was observed (Fig. 5). Of note are results from experimental day 15, 1 day after CB supplementation stopped, when the copper concentration in TRT was 20.3 times greater than that in the CTL samples (2,234 ppm in TRT and 110 ppm in CTL). This suggests residual copper-containing beads likely persist in the swine GI tract for some time after being administered. However, the values dramatically decreased after the CBs were removed for 5 days (experimental day 19), suggesting most CBs may be excreted within a few days' time. Nonetheless, copper in the TRT group feces remained higher than the control for the duration of our experiments (Fig. 5). Of additional interest is the ratio between copper content in feed and feces for both the CTL and TRT groups. Targeted copper content in feed was 440 ppm for TRT and feed itself demonstrated approximately 20-ppm Cu content (22-fold higher in TRT). Remarkably, the copper concentration in feces for TRT vs CTL was 20.3 times higher, an almost identical ratio. The results confirm CBs are effective at delivering antimicrobial copper payload to the GI tract of swine.

#### Microbiome analysis

For microbiome analysis, 30 samples were selected for sequencing of the V3–V4 region of the 16s RNA gene.

**Fig 6:**
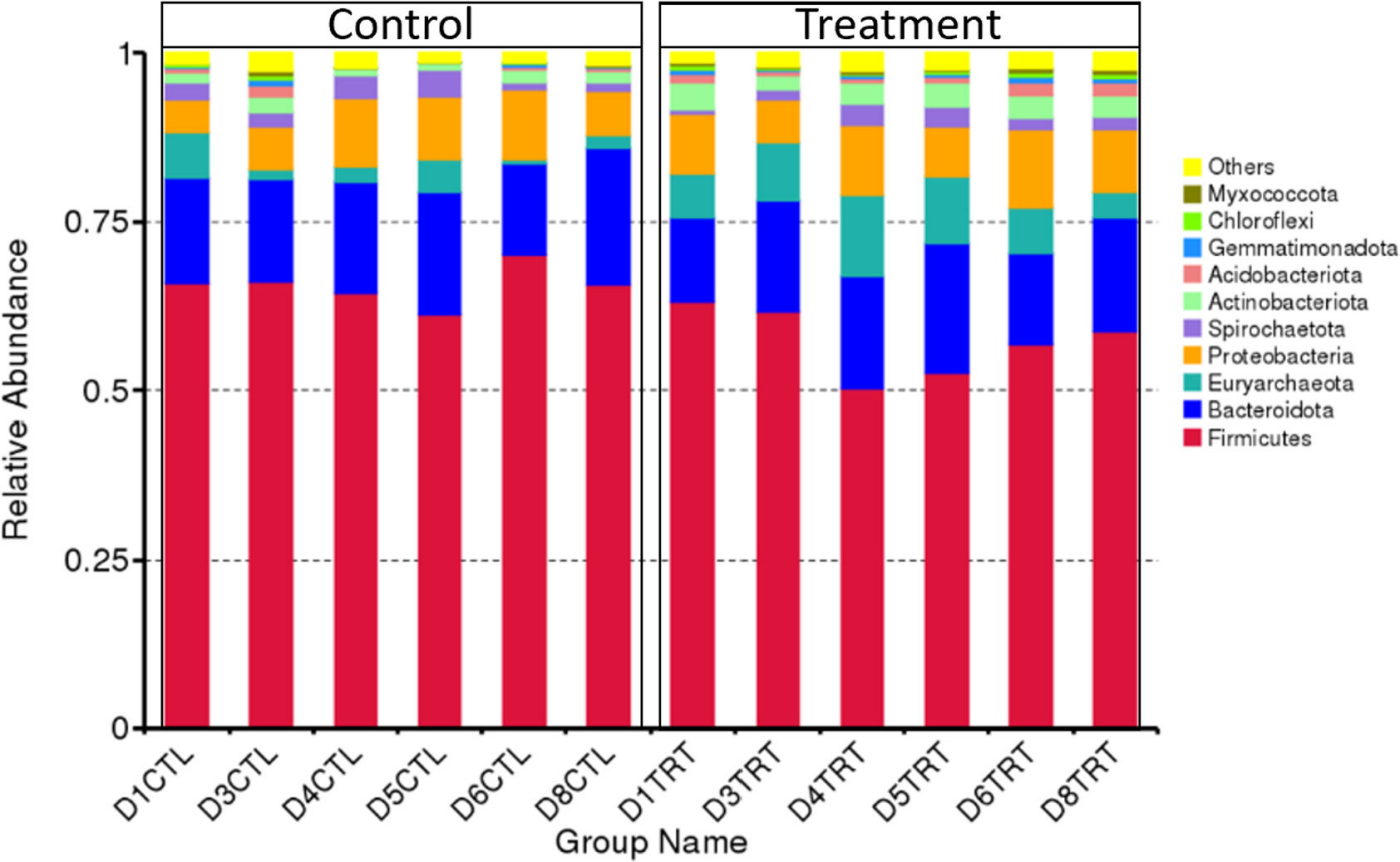
Relative abundance of taxa at the phylum level for samples grouped by treatment and day of sampling. The *Y*-axis represents relative abundance while X-axis represents group name. “Others” represents total relative abundance of the rest of the phyla besides the top ten phyla. Group name ID key goes as follows: D#: indicates sample number; TRT = pigs supplemented with copper beads, CTL = pigs do not supplement with copper beads.

A total of 39,968 species were observed in all samples analyzed. To investigate the microbial community composition in each sample, operational taxonomic units (OTUs) were obtained by clustering with 97% identity on the effective tags of all samples. To observe alpha diversities, indices between groups, Shannon indices, were calculated. The OTUs generated at 97% sequence identity were homologous species. Shannon diversity index showed higher diversity in the TRT group than the CTL group. To compare microbial communities between each pair of community samples (beta-diversity), a permutational multivariate analysis of variance was used to observe significant differences in microbial abundance of the CB group between samples. Significant differences (*P* = 0.0014) within the TRT group were first observed between samples 1 (experimental day 5 or pretreatment) and 4 (experimental day 11, after 4 days of animal receiving treatment). Consistently, the overall microbial abundance in the fecal samples continued to be significantly different (*P* = 0.0014) from sample 1 (pretreatment) to sample 6 (1 day after treatment was removed). However, no significant (*P* = 0.4) differences were observed between samples 1 (pretreatment) and 8 (6 days after treatment was removed). This indicates that the microbial abundance returned to baseline once the CB treatment was removed. Additionally, samples 3 (2 days of animals receiving treatment) and 6 (1 day after treatment was removed) showed significant differences (*P* = 0.0014), indicating that the bacterial community continued to change during copper bead supplementation. The relative abundance of taxa in the phylum is demonstrated in Fig. 6. Individual animal samples were grouped by treatment and day to facilitate observing a trend in microbial dynamics. The top 10 taxa for each group (TRT and CTL) at the phylum level were assessed. Firmicutes were the most abundant among all samples followed by Bacteroidota. In the TRT group, it was observed that on samples 4 and 5 (middle of supplementation period), there is a decrease in Firmicutes and an increase in Bacteroidota compared to the CTL group. Moreover, the TRT group had an increase in Euyarchaeota throughout the period of supplementation, and the levels remained higher after the beads were removed. Interestingly, there is an increase in Acidobacteriota in the TRT group; however, that is not seen until samples 6 and 8 (post-supplementation). This suggests that the shift in Acidobacteriota occurred toward the end of the supplementation week, and the effects remained until the last sampling point of the experiment (sample 8). Similar to the enumeration results, a clear shift in the microbiome was detected in association with the supplementation of copper beads.

**Fig 7:**
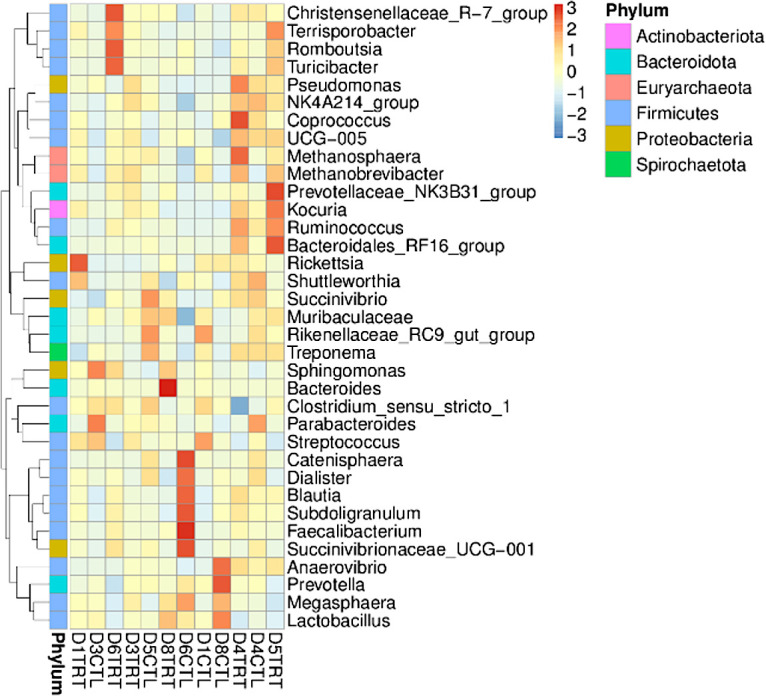
Relative abundance of taxa at the genus level for samples grouped by treatment and sampling day. The *Y*-axis represents the genus, while the *X*-axis represents group name. The heatmap scale displays the row *z* score. The absolute value of *z* represents the distance between the raw score and the mean of the standard deviation (values from −3 to 3 on the upper right corner). *z* is negative when the raw score is below the mean and vice versa in units of standard deviation. Group name ID key goes as follows: D# indicates the sample number; TRT denotes pigs supplemented with copper beads; and CTL denotes pigs not supplemented with copper beads.

A heat map was created according to the relative abundance of the top 34 genera found in all samples (Fig. 7) to check whether the samples were clustered. A decrease in the abundance of genus *Lactobacillus* (most notable at last days supplementation, in samples 5 and 6) in the TRT group can be depicted, which correlates with the LAB enumeration results. Additionally, relative abundance of *Rickettsia* appears to decrease in the TRT group throughout the supplementation of CB. However, it is important to note that the high relative abundance of *Rickettsia* is observed only in sample 1 of the TRT group and not in sample 1 of the CTL group. It is possible that the sample from the animal selected for microbiome analysis had a higher relative abundance of *Rickettsia* and CB are not solely responsible for this shift. Figure 7 shows a stronger shift in the microbial community on samples 4 and 5 (middle of bead supplementation). Sample four shows an increase in *Methanosphaera*; however, it also shows an increase in *Coprococcus* and *Pseudomonas* all from different phyla. With respect to sample 5, *Ruminococcus*, *Kocuria*, *Bacteroidales*_RF16_group, and Prevotellaceae_NK3B31_group are the most notable shifts when comparing TRT and CTL. On the other hand, when samples 1 (pretreatment) and 8 (6 days after treatment was removed) were compared, a *Lactobacillus* population increase on sample 8 was observed (higher abundance than observed in sample 1). Moreover, *Methanosphaera*, *Coprococcus*, *Pseudomonas*, *Ruminococcus*, *Kocuria*, *Bacteroidales*_RF16_group, and *Prevotellaceae*_NK3B31_group all decreased and began to have similar relative abundance than at sample 1.

## DISCUSSION

This study sought to investigate the synthesis of a novel copper-based feed additive and its effects on bacteria, both *in vitro* and *in vivo*. The supplementation of copper and other trace minerals in the diet of swine is commonly used for growth promotion and support health at an early life stage; however, it is also known that copper supplementation exhibits antimicrobial activity (14, 29–31). In monogastric species, copper is mostly absorbed in the upper gastrointestinal tract, especially in the upper part of the GI tract (18). Foodborne pathogens commonly reside in the swine intestine, potentially contaminating hides during slaughter (32). Therefore, a way to deliver copper to the intestines and target microorganisms residing in this part of the GI tract is of interest. With this in mind, copper was encapsulated in alginate beads that were further dehydrated, expecting a gradual release of copper and reaching the intestines without being absorbed in the duodenum, thus affecting microorganisms in this section of the GI tract. The increase in copper concentration in feces during supplementation indicates this goal was achieved.

The *in vitro* results have demonstrated antibacterial copper concentrations are achieved by delayed release of the ion from the encapsulated beads for 6 h when soaked in aqueous solutions. During the *in vitro* experiments with the CB treatment, copper demonstrated an antimicrobial effect against *Salmonella* over time. The beads continued to reduce *Salmonella* levels when challenged with fresh samples for >5 h. These results concur with other studies (33–35) in which copper has been assessed as an antimicrobial; however, most of the literature focuses on studying surfaces made with copper to control pathogens in food processing facilities (30). For instance, Zhu et al. (31) found that copper alloys can be a potential alternative to stainless steel for food contact surfaces as *Salmonella* Enteriditis and three copper-resistant strains of *Salmonella* Typhimurium were inactivated by copper surfaces (31). Furthermore, silver, copper oxide, and zinc oxide nanoparticles have been investigated against *Salmonella*. It was found that, although copper oxide nanoparticles reduced *Salmonella* concentrations, silver nanoparticles were more effective (36). During this study, EB and LAB were tested and enumerated *in vivo* as indicators of pathogenic microbial control in swine feces. An important finding of this study was the increase of EB and the reduction of LAB. Antimicrobial interventions for food-animals provided at preharvest level should target the reduction of certain members of EB, such as *Salmonella* or enterohemorrhagic *Escherichia coli*. The *in vitro* experiments showed a reduction of *Salmonella* after exposure to copper beads. Still, such results cannot be fully comparable with *in vivo* since those conditions did not mimic that of a swine GI tract. The presence of a particular microbiota in the pig’s gastric fluid, anaerobiosis conditions, pH, and any metabolites present may impact the effect of the treatment. Therefore, additional studies *in vitro* emulating the animals’ gastric fluids and conditions will help elucidate the appropriate conditions needed to target reductions in members of the family Enterobacteriaceae members.

Existing methods to control pathogen shedding in animals include the supplementation of direct-fed microbial (DFM), vaccination, sodium chlorate, antibiotics, and hygienic practices, among others (32, 37, 38). The addition of DFM to the swine diet can reduce the prevalence of diarrhea, improve feed conversion, nutrient utilization, growth performance, intestinal microbiota, and regulate the immune system (39, 40). On the other hand, vaccination of cattle with type III secreted proteins significantly reduced the prevalence as well as the concentration of bacteria shed in the feces (41). Therefore, future studies can include a comparison of the copper alginate beads with current preharvest interventions.

The enumeration results of this study coincide with those of Wells et al. (42), who found an increase in coliform concentration and a decrease in *Lactobacillus* spp. in swine feces after supplementation with copper sulfate (42). Several theories could explain this outcome. The bacterial cell walls of Gram-positive and Gram-negative bacteria may be responsible for the different interactions with copper. Gram-negative bacteria have a lipopolysaccharide layer which makes them less susceptible to antibacterial agents (13). It is possible that the lipopolysaccharide layer can protect Gram-negative bacteria from interacting with copper.

Conversely, when probiotics are used as a dietary supplement to control pathogenic bacteria, it is thought that introducing these microorganisms reduce foodborne pathogens through competition in the gut (43). Therefore, an alternate explanation of our results is that the inhibition of LAB caused by CB reduced competition in the gastrointestinal tract, which allowed the EB concentrations to increase. This is further supported in the data by the observed initial reduction of LAB followed by an increase in EB. Thus, we hypothesize that the increase in EB is perhaps a result of the LAB decrease.

It is important to note that copper is an essential trace nutrient for pigs (44). Therefore, bacteria may have evolved copper homeostatic control mechanisms from copper already present in copper feed prior to supplementation (45). Resistance mechanisms against copper for bacteria for both Gram-negative and Gram-positive bacteria have been explored (46–48). In *E. coli*, the expulsion of excess copper is mediated via the CopA copper ATPase and CusCFBA transporter (48). On the other hand, LAB could potentially be more susceptible to copper. Although there are mechanisms to extrude copper from the cell, Mrvčić et al. found that LABs are very sensitive to copper (47). Interestingly, microbiome results of the 34 most abundant genus in the fecal samples did not show any genus from the family of Enterobacteriaceae. Therefore, it is possible that the higher numbers do not directly indicate an increase in pathogenic bacterium that can cause foodborne illnesses in the samples.

On the other hand, fecal microbiota shifts were observed in animals supplemented with CB. Copper concentration in feces was high, indicating that the copper in the beads is not fully absorbed and is slowly released in the GI tract of swine. Other studies have demonstrated shifts in the microbiome after copper supplementation (49). Kim et al. studied fecal microbiome shifts with different types of copper (49). They found that copper sulfate decreased the abundance of *Lactobacillus* and *Megaspheara*. Additionally, Brinck et al. researched microbiome shifts and the antibiotic resistome. Their investigation discovered that microbiome shifts could not be directly linked to Cu but to growth stages in swine production and did not find evidence for copper-induced selection of antibiotic resistance genes (50). However, the studies mentioned above did not intend to use Cu supplementation as a strategy to control foodborne pathogens. Thus, further research is needed to understand the short-term effects of Cu in the swine GI tract microbiome.

The present study observed an increase in the phylum of Acidobacteriota in the later stages of CB supplementation. Since Acidobacteriota are acidophilic, a decrease in pH of the GI tract due to copper supplementation could have created conditions for Acidobacteriota to proliferate (51). *Lactobacillus* sp. has been demonstrated to decrease gastric acid production; thus, lower LAB, as found in this study, may have influenced a potential reduction of the GI tract pH (52). Additionally, an increase in the phylum Euryarcheota was observed throughout supplementation. This is further emphasized by the increase in *Methanosphaera*, a genus belonging to this phylum (53). The increase in *Methanosphaera* could be attributed to an increase in the phylum Bacteriodota, as observed in the present study. *Methanosphaera*’s main carbon source is acetate, which is highly produced by members of the phylum Bacteriodota (54). Moreover, an increase in *Ruminococcus* spp. was observed in the investigation; this organism is also known to be an acetate producer, although this genus belongs to the phylum Firmicutes (55). These results differ from those of Zhang et al. (56), who fed piglets two different concentrations of copper (low vs high) and found that there was an increase in Euryarcheota in the control group, not the group treated with either concentration of copper (56). Contrastingly, another group of researchers found an increase in *Methanosphaera* after supplementing piglets with 160 mg/kg of Cu in colon digesta, agreeing with the results from the present study (57).

Supplementing pigs with the experimental treatment neither influence animal performance (abnormal weight changes) nor lead to any observed animal health issues.

Lastly, the present research demonstrated that copper alginate beads provide an advantage in delivering copper as a supplement in pigs’ diets since the formulation facilitates the slow release of the mineral in the GI tract. Data obtained throughout the experiments clearly show evidence of the effect of copper in fecal microbial communities, as observed in microbial shifts when copper was added to the pig’s feed. As expected, the fecal microbiota rapidly returned to initial conditions when copper was removed from the diet. Nonetheless, more research is needed to elucidate factors influencing the decrease in LAB and the increase in EB. Challenge studies in which animals are exposed to pathogens and treated with CB may be useful to understand better the promise of the technology as a feed additive for swine. Furthermore, additional experiments to test treatment variations are required to identify the best dosage and exposure that aid in controlling specific targeted bacteria. Future research *in vitro* might explore how CB affects the complex interactions between microbial communities of different microorganisms within the swine monogastric system.

## Data Availability

Data are accessible through the National Center for Biotechnology Information (NCBI) (bioproject accession number PRJNA1084847).
